# The role of three interleukin 10 gene polymorphisms (− 1082 A > G, − 819 C > T, − 592 A > C) in the risk of chronic and aggressive periodontitis: a meta-analysis and trial sequential analysis

**DOI:** 10.1186/s12903-018-0637-9

**Published:** 2018-10-22

**Authors:** Hey Chiann Wong, Yuxuan Ooi, Shaju Jacob Pulikkotil, Cho Naing

**Affiliations:** 1 School of Dentistry, International Medical University, Kuala Lumpur, Malaysia; 2 Institute for Research, Development and Innovation (IRDI), International Medical University, 5700 Kuala Lumpur, Malaysia; 3 Division of Tropical Heath and Medicine, James Cook University, Townsville, Australia

## Abstract

**Background:**

Periodontitis is a major oral health problem and it is considered as one of the reasons for tooth loss in developing and developed nations. The objective of the current review was to investigate the association between *IL10* polymorphisms − 1082 A > G (rs1800896), -819C > T (rs1800871), − 592 A > C (rs1800872) and the risk of either chronic periodontitis or aggressive periodontitis.

**Methods:**

This is a meta- analysis study, following the preferred reporting items for systematic reviews and meta- analyses (PRISMA). Relevant studies were searched in the health related electronic databases. Methodological quality of the included studies were assessed using the Newcastle-Ottawa Scale. For individual studies, odds ratio (OR) and its 95%confidence interval (CI) were calculated to assess the strength of association between *IL10* polymorphisms (− 1082 A > G, -819C > T, − 592 A > C) and the risk of periodontitis. For pooling of the estimates across studies included, the summary OR and its 95% CIs were calculated with random-effects model. The pooled estimates were done under four genetic models such as the allelic contrast model, the recessive model, the dominant model and the additive model. Trial sequential analysis (TSA) was done for estimation of the required information size for this meta-analysis study.

**Results:**

Sixteen studies were identified for this review. The included studies were assessed to be of moderate to good methodological quality. A significant association between polymorphism of *IL10*–1082 A > G polymorphism and the risk of chronic periodontitis in the non-Asian populations was observed only in the recessive model (OR,1.42; 95% CI:1.11, 1.8,*I*^2^: 43%). The significant associations between − 592 A > C polymorphism and the risk of aggressive periodontitis in the non-Asian populations were observed in particular genetic models such as allele contrast (OR, 4.34; 95%CI:1.87,10.07,*I*^2^: 65%) and recessive models (OR, 2.1; 95% CI:1.16, 3.82,*I*^2^: 0%). The TSA plot revealed that the required information size for evidence of effect was sufficient to draw a conclusion.

**Conclusions:**

This meta-analysis suggested that the *IL10*–1082 A > G polymorphism was associated with chronic periodontitis CP risk in non-Asians. Thus, in order to further establish the associations between *IL1*0 (− 819 C > T, − 592 A > C) in Asian populations, future studies should include larger sample sizes with multi-ethnic groups.

**Electronic supplementary material:**

The online version of this article (10.1186/s12903-018-0637-9) contains supplementary material, which is available to authorized users.

## Background

Periodontitis is defined as an inflammatory disease of supporting tissues of teeth caused by specific microorganisms or groups of specific microorganisms, resulting in progressive destruction of the periodontal ligament and alveolar bone with periodontal pocket formation, gingival recession or both [1]. There are two forms of periodontitis such as chronic periodontitis (CP) and aggressive periodontitis (AP). Severe periodontitis can result in loosening of teeth, leading to occasional pain or discomfort and impaired mastication and eventual tooth loss [2]. Periodontitis is a major oral health problem and it is considered as one of the reasons for tooth loss in developing and developed nations.

Worldwide, the prevalence of CP in the general adult population was reported to be 30–35%, with approximately 10–15% diagnosed with severe CP [3] with geographic variation. For instance, the estimated prevalence of CP and severe periodontitis among general population in Malaysia was 48.5%, and 18.2% respectively [4]. According to the National Health and Nutrition Examination Survey (NHANES) 2009–2010, the prevalence of periodontitis and severe periodontitis of adults aged 30 years and above in the United States were 47.2% and 8.5%, respectively [5].

Although the presence of microorganisms is crucial for the clinical status, host response to bacteria triggers the secretion of pro-inflammatory mediators, leading to extra cellular matrix metabolism and bone resorption in periodontitis [6, 7]. Interleukin (*IL*)10 is an immunoregulatory cytokine and down-regulates the Th1 driven pro-inflammatory response [8]. It is produced mainly by macrophages apart from numerous other cells such as Th2 cells, dendritic cells, B-cells, monocytes, neutrophils eosinophils and mast cells [9]. Studies have shown that dysregulation of *IL10* is associated with an enhanced immunopathological response to infection as well as an increased risk for the development of many autoimmune diseases [10] and infections such as tuberculosis [11]. Although about 99% of human genes are shared across the same population, variations in sequence and single nucleotide polymorphisms (SNPs) may have significant predictive relevance. Several IL10 genes implicated to affect IL10 transcription and secretion include the three biallelic polymorphisms at position − 1082, − 819 and − 592 [12, 13]. There are molecular epidemiological studies that assessed the association between *IL10* and the risk of developing periodontitis. These individual studies, however, showed conflicting results. Such inconsistency of the results may be attributed to small sample size, insufficient statistical power and ethnic diversity of the population. Taken together, the objective of the current review was to investigate the association between polymorphisms of *IL10*–1082 A > G (rs1800896), -819C > T (rs1800871), − 592 A > C(rs1800872) and the risk of either CP or AP.

## Methods

The current study followed the preferred reporting items for systematic reviews and meta- analyses (PRISMA) [14] (Additional file 1).

### Study search

Relevant studies were searched in the health-related electronic databases of PubMed, EMBASE, web of science and google scholar, with the combination of the keywords with Boolean operators: “periodontitis” OR “chronic periodontitis” OR “aggressive periodontitis” AND “interleukin-10” OR “IL10” OR “-1082 A/G”. OR “-819 C > T” OR “-592 A > C”.

The search was limited to the studies published in English and Chinese (abstract in English) from 1968 until March 2018. The references of retrieved articles and relevant reviews were checked for any additional studies.

### Inclusion criteria

Human studies of any sample size that assessed periodontitis were included, if:

i) *IL10*–1082 A > G (rs1800896), 592 A > C (rs1800872) and/or − 819 C > T (rs1800871) were investigated.
ii) it was case-control or nested case-control design.
iii) periodontally healthy controls were included.
iv) genotype distribution in both cases and controls were available.

CP and AP in the current analysis were as defined in the primary studies. Studies, which did not meet the inclusion criteria were excluded. Studies based on family or sibling-pairs were also excluded.

### Data extraction

Three investigators (HCW, YO, CN) individually checked the titles and abstracts, and then selected the relevant full-text articles, according to the inclusion criteria. The three investigators independently extracted data, using a piloted data extraction form. The following information were collected from each study: author, publication year, country, design (e.g. hospital-based study, population-based study), the number of cases and controls, the racial descent (Asian or non-Asian), clinical types of periodontitis, genotyping method and genotype/allele distribution in both cases and controls. Any discrepancy was resolved by discussion and consensus. If allele frequency was zero, then a value of 1 was added to all allele, following Laplace approximation [15].

### Methodological quality assessment

The two investigators (HCW, YO) independently assessed the methodological quality of studies, using the Newcastle-Ottawa Scale (NOS) for quality assessment [16], with necessary modifications. The scores were based on three main aspects: the selection of the study groups (maximum 4 points: whether the case definition was adequate with independent validation; whether the patients were consecutive or obviously representative series of cases; whether the controls were population-based; whether the controls were persons without history of periodontitis); the comparability of the groups (maximum 2 points: whether cases and controls were matched for age and gender; whether cases and controls were matched for region or ethnicity); and the ascertainment of exposure (maximum 3 points: whether the ascertainment of exposure was secure record; whether the same method of ascertainment was applied for cases and controls; whether the same non-response rate was reported for both groups). A total score for each study can vary from 0 (worst) to 9 (best). Studies scoring ≤4 were regarded as low quality, while a score of ≥7 was considered as a high-quality study. Any discrepancy between the two investigators was resolved by discussion and consensus.

### Statistical analysis

Hardy-Weinberg Equilibrium (HWE) in the controls was tested, using the exact test for goodness-of-fit and *p* > 0.05 was regarded as consistent with HWE [17]. The strength of the association between *IL10* (− 1082 A > G,-819 C > T, − 592 A/C) and the risk of periodontitis (CP or AP) was estimated using odds ratio (OR) and its 95% confidence interval (CIs). Heterogeneity was statistically assessed using the *I*^2^ test. The *I*^2^ test values describes the percentage of total variation across studies that is attributable to the heterogeneity rather than chance. *I*^2^ values greater than 50% is considered as substantial heterogeneity [18]. For pooling of the estimates across studies included, the summary ORs and corresponding 95% CIs were calculated with random-effects model (The Der Simonian and Laird method) in the presence of between–study heterogeneity of the studies. Otherwise, fixed-effect model (the Mental-Haenszel method) was used. We estimated the pooled ORs and its 95% CIs in four genetic models: − 592 A > C the allelic contrast model (A vs C), the recessive model (AA vs CA + CC), the dominant model (AA+CA vs CC) and the additive model (AA vs CC). As for − 1082 A > G the allelic contrast model (A vs G), the recessive model (AA vs GA + GG), the dominant model (AA+GA vs GG) and the additive model (AA vs GG). As for − 819 C > T the allelic contrast model (T vs C), the recessive model (TT vs CT + CC), the dominant model (TT + CT vs CC) and the additive model (TT vs CC) was used. Analysis was further stratified by the clinical forms of periodontitis (CP, AP) and by ethnic decent. To assess the stability of results, the sensitivity analysis was done with leave-one-out meta-analysis by sequential omission of individual studies [19]. We assessed the publication bias by visualizing funnel plots [20, 21].

Trial sequential analysis (TSA), an approach that adjust for random error risk, was done for estimation of the required information size [22]. It is classified as ‘potentially spurious evidence of effect’, if the cumulative Z-curve did not cross the monitoring boundaries or as ‘firm evidence of effect’, if the cumulative Z-curve crossed the monitoring boundaries [23].

Meta-analysis was done with RevMan 5.3 (The Cochrane collaboration, Copenhagen) and Leave-one-out meta-analysis was with *meta* package (*metabin* command) in *R* 3.4.3 software (The *R* Foundation). TSA was done with TSA version 0.9 beta (Copenhagen Trial Unit, Centre for Clinical Intervention Research, Copenhagen).

## Results

**Fig. 1:**
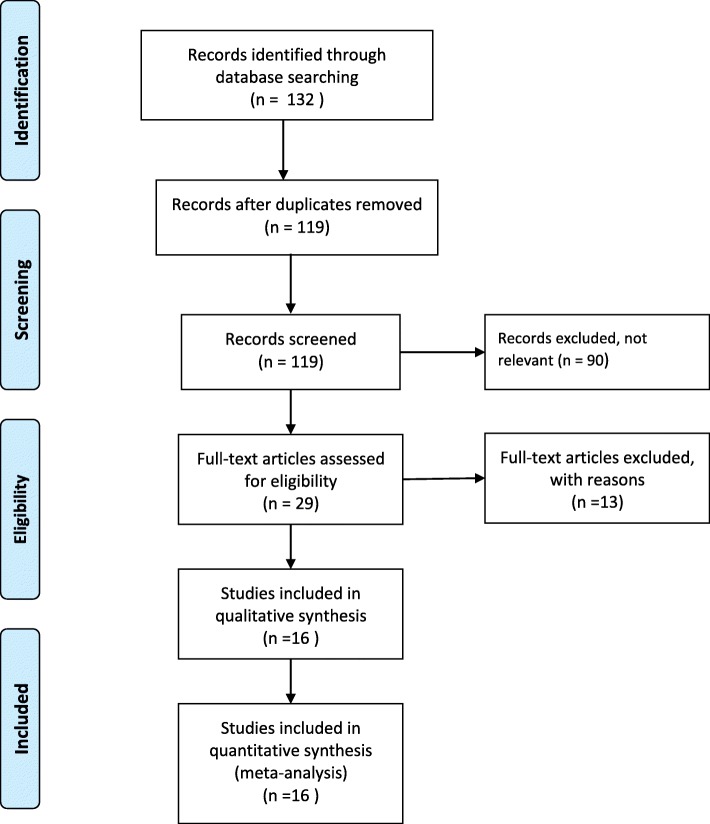
Flow chart of the study selection process

Figure 1 provides a four-phase study selection process in the present meta-analysis study. A total of 132 studies were identified in the initial search. After the title and abstract screening, 29 full- text articles were retrieved. Of these, a final of 16 articles (with 1557 cases and 1447 controls) were identified for this review [24–39]. All except one study were published in English language. One study was published in Chinese language, but provided English language abstract [27]. The studies included used different criteria for diagnosis of periodontitis such as clinical criteria (44%,7 studies), 1999 classification of periodontal diseases and conditions (25%,4 studies), clinical criteria and X-ray verification (19%, 3 studies) and criteria of the American Academy of Periodontology (13%, 2 studies).

The main characteristics of the studies identified for this review are presented in Additional file 2. Summary of the 13 excluded studies [12, 40–51] are provided in Additional file 3. These 16 studies included were conducted in nine countries such as eight single studies from China [27], Germany [29], Macedonia [33], Norway [36], Peru [34], Taiwan [30], Turkey [31] and the United Kingdom [25], three studies from Iran [26, 33, 39] and six studies from Brazil [24, 28, 30, 35, 37, 38]. Of these sixteen, six studies assessed all three candidate SNPs [24, 30, 32, 36–38] in the risk of CP/AP, while thirteen and nine studies assessed − 1082 A > G, and − 819 C > T or − 592 A > C, respectively. All these studies recruited healthy controls. All, except two studies [32, 34] were consistent with HWE. All these 16 studies were with moderate to good methodological quality, based on the NOS criteria (Additional file 4).

### Quantitative estimates

**Fig. 2:**
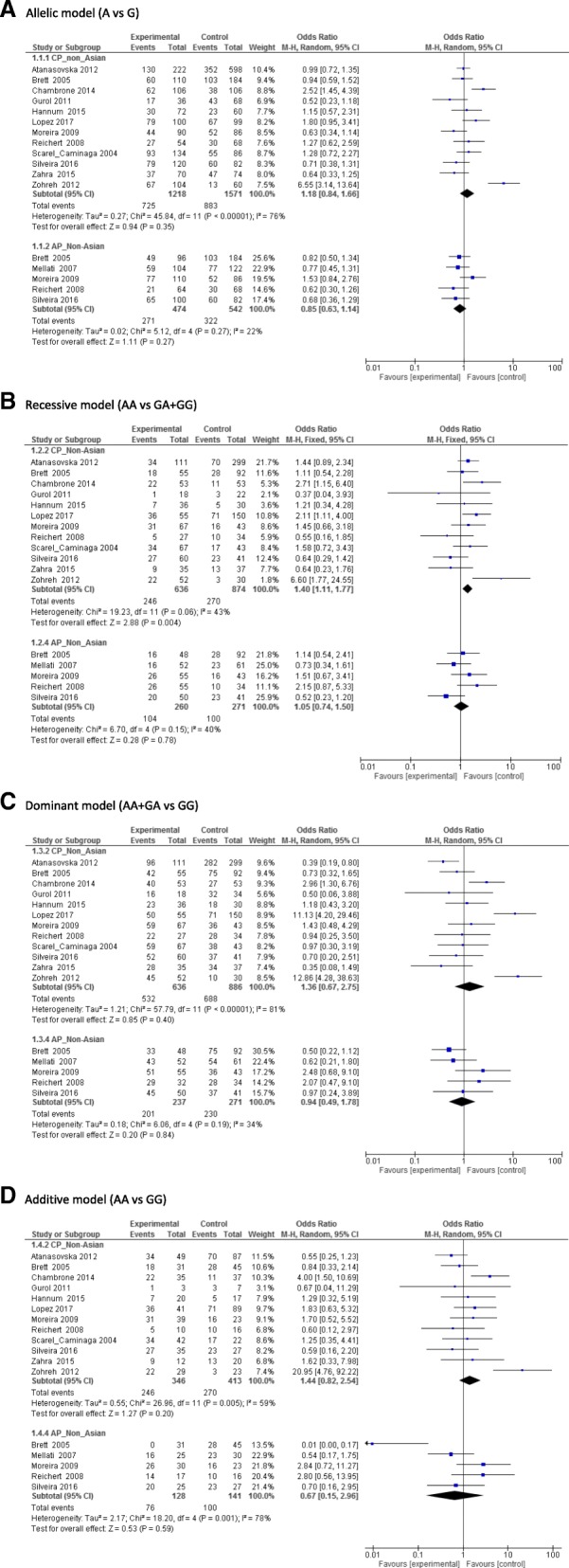
Forest plot of − 1082 A > G. **a** Allele genetic model, **b** Recessive model, **c** Dominant model, **d** additive model

**Fig. 3:**
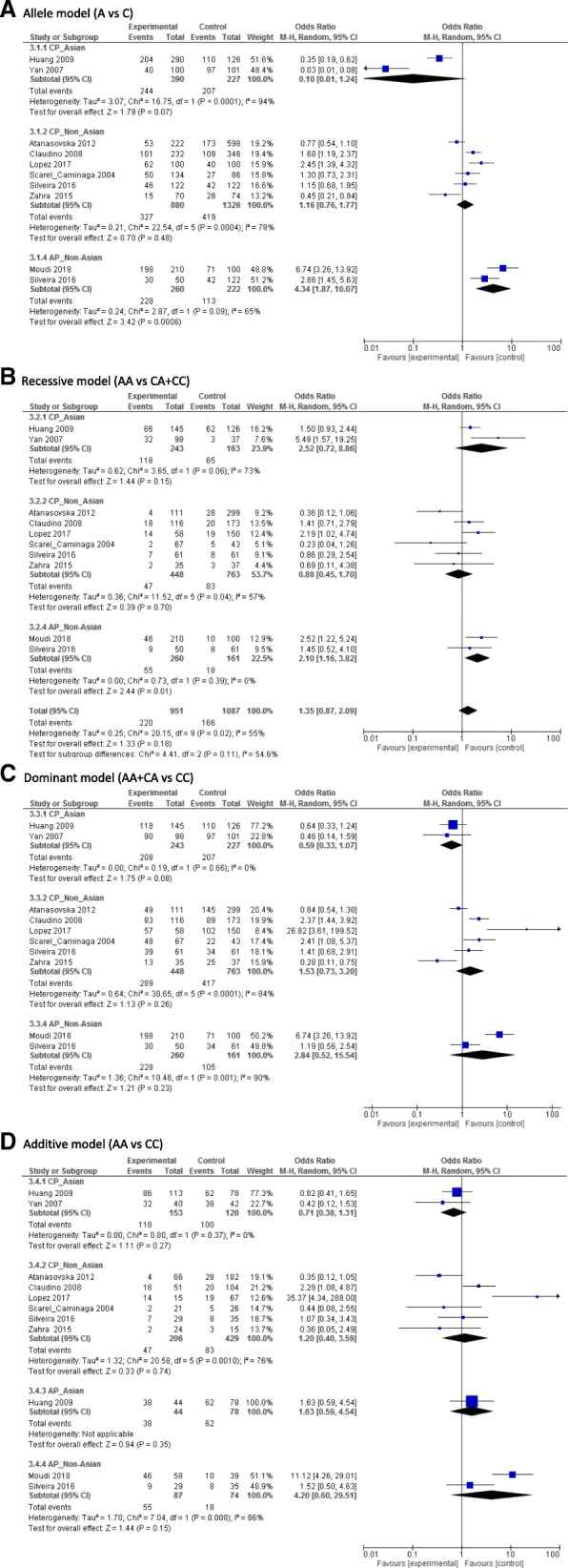
Forest plot of − 592 A > C. **a** Allele genetic model, **b** Recessive model, **c** Dominant model, **d** additive model

**Fig. 4:**
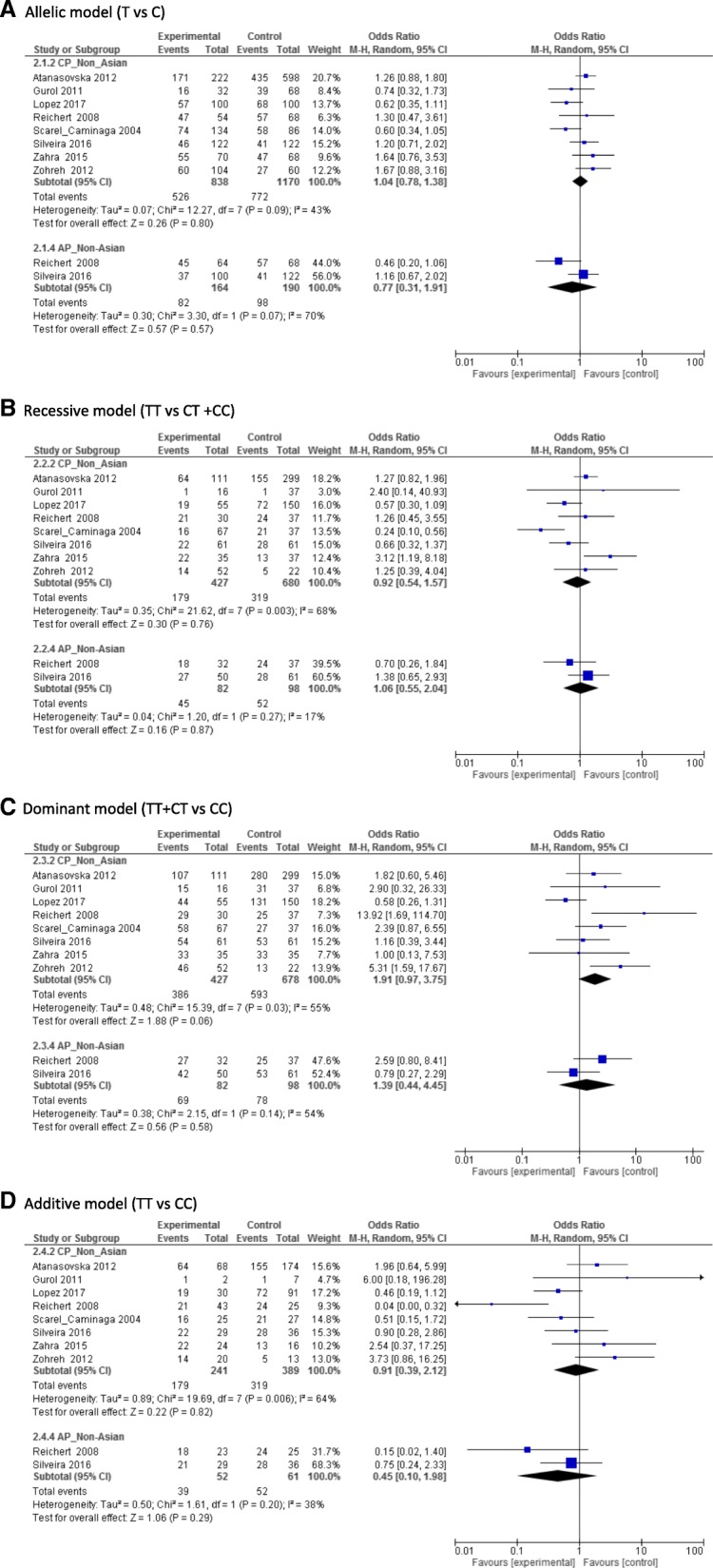
Forest plot of − 819 C > T. **a** Allele genetic model, **b** Recessive model, **c** Dominant model, **d** additive model

Overall, there was statistical significant associations between *IL10*–1082 A > G polymorphism and the risk of AP in a subgroup of the non-Asian population under the recessive model (OR,1.42;95% CI,1.11–1.8,*I*^2^:43%) (Fig. 2). For *IL10*–592 A > C, only the non-Asian populations under the allele contrast model (C vs A) (OR,4.34; 95%CI:1.87–10.07, *I*^2^:65%) and the recessive model (OR,2.1;95% CI,1.16–3.82,*I*^2^:0%) showed significant relationship between this SNP and the risk of AP (Fig. 3). For *IL10*–819C > T, there was no significant associations between this SNP and the risk of CP/AP in any population groups under any of four genetic models (Fig. 4). Summary of the evaluation of the three candidate SNPs stratified by population groups and clinical types under four genetic models are provided in Additional file 5.

### Sensitivity analysis

**Fig. 5:**
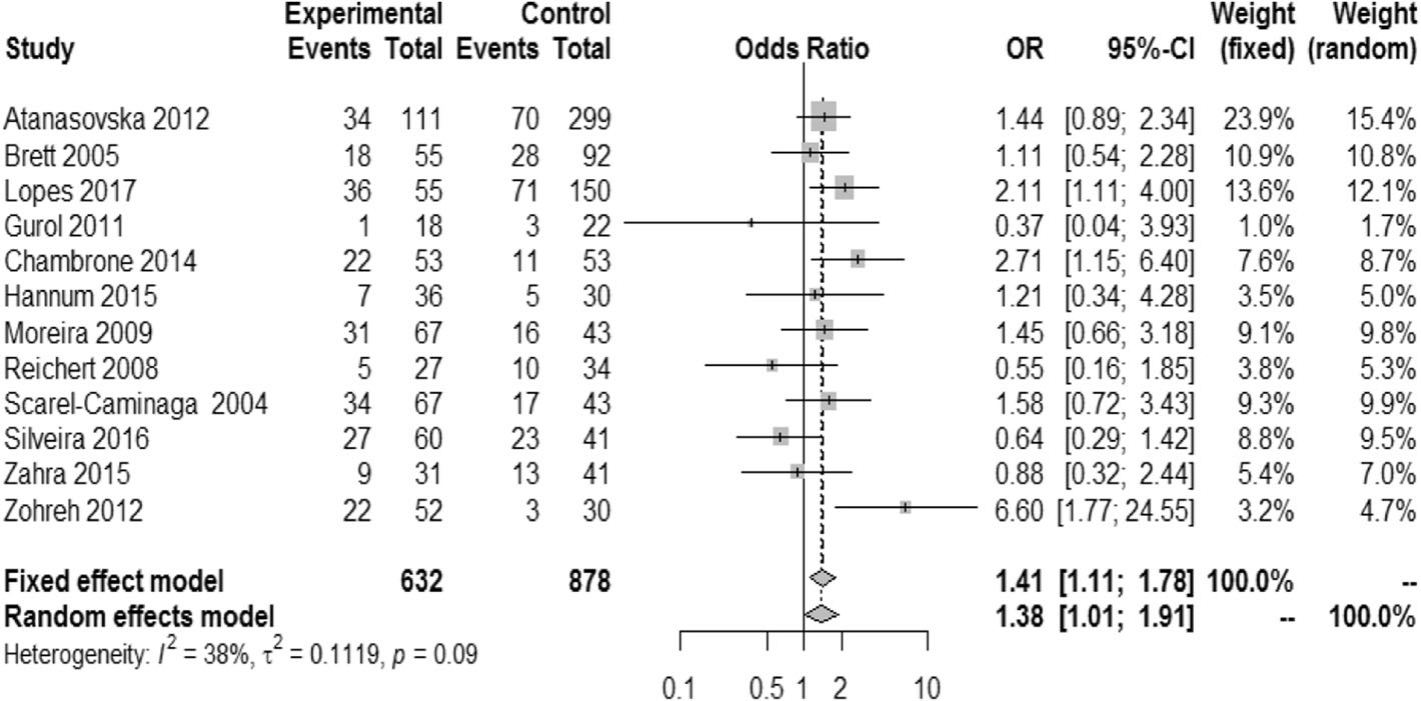
Leave-one out analysis on − 1082 A > G Allele genetic model

To assess the influence of individual data on the pooled ORs, leave-one-out meta-analysis was performed. The overall estimate of recessive model of *IL10*–1082 A > G remained statistical significance, while omitting any single study (Fig. 5). This indicates that the results are stable. This was true for all other models with the 3 candidate SNPs. As an example, a subgroup analysis stratified by diagnostic criteria was done on CP patients with − 1082 A > G under recessive model: CP patients diagnosed only by clinical criteria was significantly associated with this SNP (Additional file 6). This implied that the diagnostic criteria for CP had impact on the direction of association. Begg’s and Egger’s test showed no evidence of publication bias in all analyses concerning *IL10* (− 1082 A > G, − 819 C > T, 592C > A) polymorphisms. An example illustration with *IL10*–1082 A > G under dominant model is shown in Additional file 7.

### TSA

We performed TSA with studies on *IL10*–1082 A > G in CP that followed HWE. We used an overall 5% risk of a type I error and 20% risk of a type II error (power of 80%). The cumulative z-curve crossed the traditional boundary and the trial sequential monitoring boundary and reached the required information size, suggesting there is no need for more evidence to establish additional study of − 1082 A > G in CP among the non-Asian population (Additional file 8).

## Discussion

The current study provides evidence on the association between *IL10* (**−** 1082 A > G, − 819 C > T, − 592 A > C) and the risk of CP/AP, and the major observations are as follows;

i. A significant association between − 1082 A > G polymorphism and the risk of CP in the non-Asian populations was observed only in the recessive model.
ii. In subgroups with ethnicity, the significant associations between − 592 C > A polymorphism and the risk of AP in the non-Asian populations were observed in particular genetic models such as allele contrast and recessive models.
iii. The TSA plot revealed that the required information size for evidence of effect was sufficient to draw a firm conclusion.

The numbers of inflammatory leucocytes that express the anti-inflammatory IL10- cytokine are much more widely distributed than those that express the pro-inflammatory IL6*-* cytokine and TNF-a- cytokine [52]. The pooled analysis of 11 studies in this review showed a significant association between -1082A > G and the risk of CP in the non-Asian population under recessive model and this estimate was stable even with sensitivity analysis. Therefore, the findings suggest a possible role of racial difference in genetic backgrounds. Genotypic differences in cytokine genes are differently inherited to ethnically different populations [24]. The lack of statistical significance in the remaining genetic models might partly be due to the relatively small sample size. This was indirectly supported by an earlier review on this SNP, which included eight studies on patients with CP had reported no significance association with -1082A > G under any of four genetic models [53]. Another meta-analysis in this field, which included nine case-control studies had also reported no significance association with -1082A > G under any genetic models [54]. In subsequent analysis of patients with AP, no significant association was found with this SNP and this also might partly be related to limited size of samples as relatively few number of studies were assessed for this clinical form.

A significant association between − 592 A > C polymorphism and the risk of AP in allele contrast and recessive model indicated the prominent role of AA in the risk development. Although environmental factors are considered to be important in the establishment of periodontitis, individuals reared or living in similar environments may manifest significantly different disease patterns [55]. The pooled results showed no significant association between − 819 C > T and the risk of CP/AP in the non-Asian population under all four genetic models could also be related to ethnical homogeneity of the studied populations [34], the limited size of samples or different in selection criteria of patients/controls. The − 819 C > T SNP lies within a DNA motif forming a putative oestrogen responsive element [56]. Hence, the dominant proportion of females or males in the primary studies was likely to affect the lack of associations in this review.

The reason why different association was observed in different SNPs remained unclear. One possible explanation is the differences in the frequencies of the polymorphisms among diverse ethnicity, which may partly give rise to heterogeneity. Moreover, the complex nature of the disease, which is the result of an interplay between immunological, microbiological and environmental factors, may be the reason for that no difference [40]*.* Smoking habit represents an additional factor involved in periodontal disease progression that could mask the effects of *IL10* SNPs on periodontitis outcome [44]. Additionally, gene-gene interactions along with environmental factors could also contribute to the complexity of genetic effect. Hence, variation in single genetic locus is often insufficient to predict risk of disease. For instance, there might be some extent of interaction with *IL10* (− 1082 A > G, − 819 C > T, 592 C > A) and other genes (gen-gen interaction/synergism).

It is postulated that polymorphisms in genes that codify mediators involved in the upstream positions of inflammatory-immune response pathways (such as IL-10 cytokine), which modulate a broad range of factors, may be relevant to periodontitis outcome [28, 57].

Genotypic differences in cytokine genes are differently inherited to ethnically different populations [24]. Individuals who are high producers of IL-10 cytokine might be more protected against CP due to its anti-inflammatory role. Therefore, a genetically determined increase of anti-inflammatory IL-10 cytokine would down regulate the immune response against periodontopathogenic bacteria [41, 51].

On the other side, polymorphisms in downstream genes (such as metalloproteinase and osteoclastogenesis), whose products present a narrow action in the pathways involved in periodontal tissue destruction, would play a minor role (or even do not play a significant role) in the development of periodontal diseases [28]. For the development of AP, low interleukin-10 levels lead to an enhanced release of pro-inflammatory cytokines, such as tumor necrosis factor-alpha, which have been implicated in alveolar bone loss [29, 58].

### Study limitations

There are some limitations in the present study. Only two studies included in this meta-analysis (11.7%) were from the Asia population. A small sample size in the Asian population subgroup analyses may not be the representative of the population. Hence, a selection bias with geographical imbalance is a concern. Our analysis was done with the unadjusted raw data provided in the primary studies, in which patients might have some common factors (i.e. age, gender, diets, smoking habits, diabetes). An earlier meta-analysis had highlighted that the inclusion of both smoking and non-smoking subjects in the primary studies can be an additional source of variability [54]. Stratified analysis by age group, smoking habits, oral hygiene habits or presence of comorbidity were not possible due to limited data. Hence, the findings could be confounded with these common factors. A stratification analysis on − 1082 A > G in the CP patients documented that the estimates could vary with the type of diagnosis criteria. If cases and controls have been genotyped in separate batches in the primary studies, differential misclassification of exposure is a concern. The sample sizes in most of the included studies were relatively small and were under power to detect statistically significant differences between the cases and controls. Meta-analysis, however, is a retrospective synthesis of published studies and power analysis is not applicable and type II errors are expected to be less common in a meta-analysis than in single studies [18, 19]. The manifestation of many systemic diseases which may lead to compromised host function should also be considered. For example, diabetes and rheumatoid arthritis are examples of diseases which may have a genetic component and may have enhanced periodontal breakdown as a secondary feature. In the presence of publication bias in this analysis, non-published studies and/or studies in other language might have been missed in the current review. Moreover, there might be some extent of interaction with *IL10* (− 1082 A > G, − 819 C > T, *IL10* 592 A > C) and other genes (gen-gen interaction/synergism). Hence, findings in the current meta-analysis should be interpreted with caution.

### Implications

Interventions with the manipulation of anti-inflammatory cytokines have been suggested as adjuvants for treating periodontal disease. The efficacy of anti-cytokine biotherapies in patients with inflammatory diseases is a proof that blocking the effects of a cytokine can slow down the disease process [59]. Investigating the association of the *IL10* gene polymorphisms with periodontitis susceptibility may promote our understanding of its pathogenesis and explain individual differences in the risk.

## Conclusions

Findings suggest that the *IL10*–1082 A > G polymorphism was associated with CP risk in non- Asians. To further establish the associations between *IL10* (819 C > T, 592 A > C), future studies with larger sample sizes and multi-ethnic sample groups more with Asian population are required.

## Additional files

Additional file 1: PRISMA Checklist. (DOC 60 kb)

Additional file 2: Characteristics of the included studies. (DOC 78 kb)

Additional file 3: The excluded studies. (DOC 34 kb)

Additional file 4: The methodological quality of studies. (DOC 45 kb)

Additional file 5: Summary of the pooled estimates for the three polymorphisms. (DOC 103 kb)

Additional file 6: Subgroup analysis of the studies on *IL10*–1082 A > G in the recessive model. (PDF 105 kb)

Additional file 7: Funnel plot of studies on *IL10*–1082 A > G in the recessive model. (PDF 84 kb)

Additional file 8: Trial sequential analysis of studies on *IL10*–1082 A > G in chronic periodontitis. (PDF 211 kb)

## Abbreviations

AP: Aggressive periodontitis
CI: Confidence interval
CP: Chronic periodontitis
HWE: Hardy-Weinberg Equilibrium
IL: Interleukin
OR: Odds ratio
PRISMA: Preferred reporting items for systematic reviews and meta-analyses
SNP: Single nucleotide polymorphisms
TSA: Trial sequential analysis
