# Supplementary material for: The role of three interleukin 10 gene polymorphisms (− 1082 A > G, − 819 C > T, − 592 A > C) in the risk of chronic and aggressive periodontitis: a meta-analysis and trial sequential analysis
Source: BMC Oral Health. 2018 Oct 22;18:171. doi: 10.1186/s12903-018-0637-9 (PMC6198364; doi:10.1186/s12903-018-0637-9)
Supplement: Supplementary file 2 — Characteristics of the included studies. (DOC 78 kb) [file 12903_2018_637_MOESM2_ESM.doc]

Additional File 2. Characteristics of the included studies

A) Studies on the relationship between IL-10- 1082 A>G and periodontitis

| Study | Year | Country | Population group | Study design | Diagnostic criteria | Cases  (GG/GA/AA) | Controls  (GG/GA/AA) | HWE  (p value) | Clinical Types |
| --- | --- | --- | --- | --- | --- | --- | --- | --- | --- |
| Atanasovska-Stojanovska  et al. | 2012 | Macedonia | Non- Asian | Case-control | clinical & X-ray^§^ | 15/62/34 | 17/212/70 | 0 | CP |
| Lopes et al. | 2017 | Brazil | Non- Asian | Case-control | clinical | 5/14/36 | 18/61/71 | 0.387 | CP |
| Silveira et al. | 2016 | Brazil | Non- Asian | Cross-sectional | AAP | 13/50/47 | 4/14/23 | 0.404 | CP & AP |
| Zahra et al. | 2015 | Norway | Non- Asian | Case-control | 1999  classification^¶^ | 7/19/9 | 3/21/13 | 0.172 | CP |
| Hu et al. | 2009 | Taiwan | Han Chinese | Case-control | clinical | 0/18/192 | 0/11/115 | 0.608 | CP & AP |
| Reichert et al. | 2008 | Germany | Non- Asian | Case-control | clinical & X-ray^§^ | 8/32/19 | 6/18/10 | 0.667 | CP & AP |
| Scarel-Caminaga et al. | 2004 | Brazil | Non- Asian | Case-control | clinical | 8/25/34 | 5/21/17 | 0.317 | CP |
| Zohreh et al. | 2012 | Iran | Non- Asian | Case-control | clinical | 7/23/22 | 20/7/3 | 0.087 | CP |
| Gurol et al. | 2011 | Turkey | Non- Asian | Case-control | 1999  classification^¶^ | 2/15/1 | 2/21/11 | 0.056 | CP |
| Hannum et al. | 2015 | Brazil | Non- Asian | Case-control | clinical | 13/16/7 | 12/13/5 | 0.648 | CP |
| Chambrone et al. | 2014 | Peru | Non- Asian | Cross-sectional | clinical | 13/18/22 | 26/16/11 | 0.012 | CP |
| Moreira et al. | 2009 | Brazil | Non- Asian | Cross-sectional | 1999  classification^¶^ | 12/53/57 | 7/20/16 | 0.859 | CP & AP |
| Mellati et al. | 2007 | Iran | Khorasanian | Case-control | clinical & X-ray^§^ | 9/27/16 | 7/31/23 | 0.475 | gAP |
| Brett et al. | 2005 | UK | Non- Asian | Case-control | clinical | 28/41/34 | 17/47/28 | 0.726 | CP& AP |

AAP: American Academy of Periodontology

; AP = aggressive periodontitis; gAP = generalised aggressive periodontitis; clinical criteria: clinical attachment loss, probing depth, bleeding on probing & plaque control record; CP= chronic periodontitis; ^¶^:1999 classification of periodontal diseases and conditions; ^§^clinical & X-ray verification

B) Studies on the relationship between IL10-819 C/T and periodontitis

| Study | Year | Country | Population group | Study design | Diagnostic criteria | Cases  (CC/CT/TT) | Controls  CC/CT/TT) | HWE (p value) | Clinical Types |
| --- | --- | --- | --- | --- | --- | --- | --- | --- | --- |
| Atanasovska-Stojanovska  et al. | 2012 | Macedonia | Non- Asian | Case-control | clinical & X-ray^§^ | 64/43/4 | 155/125/19 | 0.348 | CP |
| Lopes et al. | 2017 | Brazil | Non- Asian | Case-control | clinical | 19/25/11 | 72/59/19 | 0.216 | CP |
| Silveira et al. | 2016 | Brazil | Non- Asian | Cross-sectional | AAP | 43/53/15 | 28/25/8 | 0.524 | CP & AP |
| Zahra et al. | 2015 | Norway | Non- Asian | Case-control | 1999  classification^¶^ | 22/11/2 | 13/21/3 | 0.172 | CP |
| Hu et al. | 2009 | Taiwan | Han Chinese | Case-control | clinical | 22/74/114 | 12/55/59 | 0.874 | CP & AP |
| Reichert et al. | 2008 | Germany | Non- Asian | Case-control | clinical & X-ray^§^ | 38/16/5 | 23/11/0 | 0.26 | CP & AP |
| Scarel-Caminaga et al. | 2004 | Brazil | Non- Asian | Case-control | clinical | 16/42/9 | 21/16/6 | 0.317 | CP |
| Zohreh et al. | 2012 | Iran | Non- Asian | Case-control | clinical | 14/32/6 | 5/17/8 | 0.428 | CP |
| Gurol et al. | 2011 | Turkey | Non- Asian | Case-control | 1999  classification^¶^ | 1/14/1 | 0/29/5 | 0.00002 | CP |

*AP = aggressive periodontitis; CP= chronic periodontitis;* Clinical criteria: clinical attachment loss, probing depth, bleeding on probing & plaque control record

C) Studies on the relationship between IL10-592 C /A and periodontitis

| Study | Year | Country | Population group | Study design | Diagnostic criteria | Cases  (CC/CA/AA) | Controls  (CC/CA/AA) | HWE  (p value) | Clinical Types |
| --- | --- | --- | --- | --- | --- | --- | --- | --- | --- |
| Atanasovska-Stojanovska  et al. | 2012 | Macedonia | Non- Asian | Case-control | clinical & X-ray^§^ | 62/45/4 | 154/117/28 | 0.403 | CP |
| Lopes et al. | 2017 | Brazil | Non- Asian | Case-control | clinical | 0/42/13 | 48/83/19 | 0.067 | CP |
| Silveira et al. | 2016 | Brazil | Non- Asian | Cross-sectional | AAP | 42/53/16 | 27/26/8 | 0.662 | CP & AP |
| Zahra et al. | 2015 | Norway | Non- Asian | Case-control | 1999  classification^¶^ | 22/11/2 | 12/22/3 | 0.108 | CP |
| Hu et al. | 2009 | Taiwan | Han Chinese | Case-control | clinical | 33/53/124 | 16/48/62 | 0.175 | CP & AP |
| Moudi et al. | 2018 | Iran | Non-Asian | Case-control | 1999  classification^¶^ | 12/152/46 | 10/61/29 | 0.008 | CP |
| Yan et al. | 2007 | China | Asian | Case-control | clinical | 8/58/32 | 4/59/38 | 0.001 | CP |
| Scarel-Caminaga et al. | 2004 | Brazil | Non- Asian | Case-control | clinical | 19/46/2 | 21/17/5 | 0.589 | CP |
| Claudino et al. | 2008 | Brazil | Non- Asian | Case-control | AAP | 33/65/18 | 84/69/20 | 0.319 | CP |

*AP = aggressive periodontitis; CP= chronic periodontitis;* Clinical criteria: clinical attachment loss, probing depth, bleeding on probing & plaque control record
