# Supplementary material for: The role of three interleukin 10 gene polymorphisms (− 1082 A > G, − 819 C > T, − 592 A > C) in the risk of chronic and aggressive periodontitis: a meta-analysis and trial sequential analysis
Source: BMC Oral Health. 2018 Oct 22;18:171. doi: 10.1186/s12903-018-0637-9 (PMC6198364; doi:10.1186/s12903-018-0637-9)
Supplement: Supplementary file 3 — The excluded studies. (DOC 34 kb) [file 12903_2018_637_MOESM3_ESM.doc]

Additional File 3: The excluded studies

| Study, year of publication | Reference no. | Main reason |
| --- | --- | --- |
| Karhukorpi, 2001 | 12 | Not an association study |
| Gonzales, 2002 | 41 | No genotype frequency |
| Babel, 2006 | 42 | Insufficient genotype frequency |
| Sumer, 2007 | 43 | Not with the targeted SNP |
| Tervonen, 2007 | 44 | No healthy control group |
| Cullinan,2008 | 45 | Insufficient data provided |
| Kobayashi, 2009 | 46 | No healthy control group |
| Laine, 2010 | 47 | Not with the targeted genes |
| Scapoli, 2012 | 48 | Not with the targeted gene |
| Crena, 2015 | 49 | Not with the targeted SNP |
| Pirim Gorgun, 2017 | 50 | Not with the targeted SNP |
| Tettamanti, 2017 | 51 | Not provided genotype frequency |
| Token, 2018 | 52 | Not with the targeted S`NP |
