# Supplementary material for: The role of three interleukin 10 gene polymorphisms (− 1082 A > G, − 819 C > T, − 592 A > C) in the risk of chronic and aggressive periodontitis: a meta-analysis and trial sequential analysis
Source: BMC Oral Health. 2018 Oct 22;18:171. doi: 10.1186/s12903-018-0637-9 (PMC6198364; doi:10.1186/s12903-018-0637-9)
Supplement: Supplementary file 4 — The methodological quality of studies. (DOC 45 kb) [file 12903_2018_637_MOESM4_ESM.doc]

Additional File 4. The methodological quality of studies

| Study | Selection | Comparability | Exposure | Total |
| --- | --- | --- | --- | --- |
| Atanasovska-Stojanovska et al. 2012 | 3 | 2 | 3 | 8 |
| Lopes et al, 2017 | 4 | 2 | 3 | 9 |
| Silveira et al, 2016 | 3 | 2 | 3 | 8 |
| Zahra et al, 2015 | 3 | 2 | 3 | 8 |
| Hu et al, 2009 | 3 | 2 | 3 | 8 |
| Reichert et al, 2008 | 3 | 2 | 3 | 8 |
| Scarel-Caminaga et al, 2004 | 3 | 2 | 3 | 8 |
| Zohreh et al, 2012 | 3 | 0 | 3 | 6 |
| Ceyda et al, 2011 | 3 | 0 | 3 | 6 |
| Hannum et al, 2015 | 4 | 2 | 3 | 6 |
| Chambrone et al, 2014 | 3 | 0 | 3 | 6 |
| Moreira et al, 2009 | 3 | 2 | 3 | 6 |
| Mellati et al, 2007 | 3 | 2 | 3 | 6 |
| Brett et al, 2005 | 3 | 1 | 3 | 6 |
| Claudino et al, 2008 | 3 | 2 | 3 | 6 |
| Moudi et al, 2018 | 4 | 2 | 3 | 9 |
| Yan et al, 2007 | 3 | 0 | 3 | 6 |

Based on the Newcastle-Ottawa Scale (NOs) criteria. A total score for each study can vary from 0 (worst) to 9 (best).
