# Supplementary material for: The role of three interleukin 10 gene polymorphisms (− 1082 A > G, − 819 C > T, − 592 A > C) in the risk of chronic and aggressive periodontitis: a meta-analysis and trial sequential analysis
Source: BMC Oral Health. 2018 Oct 22;18:171. doi: 10.1186/s12903-018-0637-9 (PMC6198364; doi:10.1186/s12903-018-0637-9)
Supplement: Supplementary file 5 — Summary of the pooled estimates for the three polymorphisms. (DOC 103 kb) [file 12903_2018_637_MOESM5_ESM.doc]

Additional File 5. Summary of the pooled estimates for the three polymorphisms

| Description | Total studies | Participants | Odds ratio (95% CI) | *I*^2^ value |
| --- | --- | --- | --- | --- |
| -1082 A>G |  |  |  |  |
| Allele genetic model | 13 |  |  |  |
| 1 CP_Non_Asian | 12 | 2789 | 1.18 [0.84, 1.69] | 76% |
| 2 AP_Non-Asian | 5 | 1016 | 0.85 [0.63, 1.14] | 22% |
| 3 AP_Asian | 1 | 382 | 1.14 [0.39, 3.36] |  |
| 4 CP_Asian | 1 | 542 | 0.97 [0.43, 2.21] |  |
| Recessive genetic model | 13 |  |  |  |
| 1 CP_Asian | 1 | 277 | 0.99 [0.45, 2.17] |  |
| 2 CP_Non-Asian | 12 | 1510 | **1.4 [1.11, 1.77]** | 43%, |
| 3 AP_Asian | 1 | 196 | 1.14 [0.41, 3.15] |  |
| 4 AP_Non_Asian | 5 | 531 | 1.05 [0.74, 1.5] | 40% |
| Dominant genetic model | 13 |  |  |  |
| 1. CP_Asian | 1 | 277 | 1.15 [0.07, 18.55] |  |
| 2. CP_Non_Asian | 12 | 1522 | 1.36 [0.67, 2.75] | 81% |
| 3. AP_Asian | 1 | 197 | 0.52 [0.03, 8.5] |  |
| 4. AP_Non-Asian | 5 | 508 | 0.94 [0.49, 1.78] | 34% |
| Additive genetic model | 13 |  |  |  |
| 1. CP_Asian | 1 | 251 | 1.15 [0.07, 18.54] |  |
| 2. CP_Non-Asian | 12 | 759 | 1.44 [0.82, 2.54] | 59% |
| 3. AP_Asian | 1 | 179 | 0.53 [0.03, 8.55] |  |
| 4. AP_Non_Asian | 5 | 269 | 0.67 [0.15, 2.96] | 78% |
| -819C>T |  |  |  |  |
| Allele genetic model | 9 |  |  |  |
| 1 CP_Asian | 1 | 542 | 0.89 [0.62, 1.29] |  |
| 2 CP_Non_Asian | 8 | 2008 | 1.04 [0.78, 1.38] | 43% |
| 3 AP_Asian | 1 | 382 | 0.78 [0.48, 1.24] |  |
| 4 AP_Non-Asian | 2 | 354 | 0.77 [0.31, 1.91] | 70% |
| Recessive genetic model | 9 |  |  |  |
| 1 CP_Asian | 1 | 271 | 1.18 [0.53, 2.60] |  |
| 2 CP_Non_Asian | 8 | 1107 | 0.92 [0.54, 1.57] | 68% |
| 3 AP_Asian | 1 | 191 | 0.97 [0.35, 2.70] |  |
| 4 AP_Non-Asian | 2 | 180 | 1.06 [0.55, 2.04] | 17% |
| Dominant genetic model | 9 |  |  |  |
| 1 CP_Asian | 1 | 271 | 0.78 [0.48, 1.25] |  |
| 2 CP_Non_Asian | 8 | 1105 | 1.91 [0.97, 3.75] | 55% |
| 3 AP_Asian | 1 | 191 | 0.67 [0.36, 1.22] |  |
| 4 AP_Non-Asian | 2 | 180 | 1.39 [0.44, 4.45] | 54% |
| Additive genetic model | 9 |  |  |  |
| 1 CP_Asian | 1 | 139 | 1.51 [0.66, 3.49] |  |
| 2 CP_Non_Asian | 8 | 630 | 0.91 [0.38, 2.12] | 64% |
| 3 AP_Asian | 1 | 99 | 1.34 [0.45, 4.01] |  |
| 4 AP_Non-Asian | 2 | 113 | 0.45 [0.1, 1.88] | 38% |
| -592A>C |  |  |  |  |
| Allele genetic model | 9 |  |  |  |
| 1 CP_Asian | 2 | 617 | 0.1 [0.01, 1.24] | 94% |
| 2 CP_Non_Asian | 6 | 2206 | 1.16 [0.76, 1.77] | 78% |
| 3 AP_Asian | 1 | 191 | 1.43 [0.53, 3.85] |  |
| 4 AP_Non-Asian | 2 | 482 | **4.34 [1.87, 10.07]** | 65% |
| Recessive genetic model | 9 |  |  |  |
| 1 CP_Asian | 2 | 406 | 2.52 [0.7, 8.86] | 73% |
| 2 CP_Non_Asian | 6 | 1211 | 0.88 [0.45, 1.7] | 57% |
| 3 AP_Asian | 1 | 191 | 1.45 [0.79, 2.66] |  |
| 4 AP_Non-Asian | 2 | 421 | **2.1[1.16, 3.82]** | 0% |
| Dominant genetic model | 9 |  |  |  |
| 1 CP_Asian | 2 | 470 | 0.59 [0.33, 1.07] |  |
| 2 CP_Non_Asian | 6 | 1211 | 1.53 [0.73, 3.20] | 84% |
| 3 AP_Asian | 1 | 191 | 1.43 [0.53, 3.85] |  |
| 4 AP_Non-Asian | 2 | 421 | 2.84 [0.52, 15.54] | 90% |
| Additive genetic model | 9 |  |  |  |
| 1 CP_Asian | 2 | 273 | 0.7 [0.38, 1.3] | 0% |
| 2 CP_Non_Asian | 6 | 635 | 1.2 [0.4, 3.5] | 76% |
| 3 AP_Asian | 1 | 122 | 1.63 [0.59, 4.54] |  |
| 4 AP_Non-Asian | 2 | 161 | 4.2 [0.6, 29.5] | 86% |

Bold indicates the significant association.
