# Supplementary material for: The role of three interleukin 10 gene polymorphisms (− 1082 A > G, − 819 C > T, − 592 A > C) in the risk of chronic and aggressive periodontitis: a meta-analysis and trial sequential analysis
Source: BMC Oral Health. 2018 Oct 22;18:171. doi: 10.1186/s12903-018-0637-9 (PMC6198364; doi:10.1186/s12903-018-0637-9)
Supplement: Supplementary file 6 — Subgroup analysis of the studies on IL10–1082 A > G in the recessive model. (PDF 105 kb) [file 12903_2018_637_MOESM6_ESM.pdf]

Additional File 6. Recessive model of -1082 A> G in non-Asian patients stratified by diagnostic criteria

Study or Subgroup	Experimental Events	Experimental Total	Control Events	Control Total	Weight	Odds Ratio M-H, Fixed, 95% CI
**5.1.1 Clinical**						
Brett 2005	18	55	28	92	27.9%	1.11 [0.54, 2.28]
Chambrone 2014	22	53	11	53	12.7%	2.71 [1.15, 6.40]
Hannum 2015	7	36	5	30	8.7%	1.21 [0.34, 4.28]
Lopez 2017	36	55	71	150	26.1%	2.11 [1.11, 4.00]
Scarel_Caminaga 2004	34	67	17	43	20.2%	1.58 [0.72, 3.43]
Zohreh 2012	22	52	3	30	4.3%	6.60 [1.77, 24.55]
**Subtotal (95% CI)**		**318**		**398**	**100.0%**	**1.92 [1.37, 2.67]**
Total events	139		135			
Heterogeneity: Chi² = 7.08, df = 5 (P = 0.21); I² = 29%						
Test for overall effect: Z = 3.82 (P = 0.0001)						
**5.1.2 Clinical and X-ray**						
Atanasovska 2012	34	111	70	299	78.5%	1.44 [0.89, 2.34]
Reichert 2008	5	27	10	34	21.5%	0.55 [0.16, 1.85]
**Subtotal (95% CI)**		**138**		**333**	**100.0%**	**1.25 [0.80, 1.96]**
Total events	39		80			
Heterogeneity: Chi² = 2.12, df = 1 (P = 0.15); I² = 53%						
Test for overall effect: Z = 0.98 (P = 0.33)						
**5.1.3 AAP**						
Silveira 2016	27	60	23	41	100.0%	0.64 [0.29, 1.42]
**Subtotal (95% CI)**		**60**		**41**	**100.0%**	**0.64 [0.29, 1.42]**
Total events	27		23			
Heterogeneity: Not applicable						
Test for overall effect: Z = 1.09 (P = 0.27)						
**5.1.4 1999 classification**						
Gurol 2011	1	18	3	22	12.2%	0.37 [0.04, 3.93]
Moreira 2009	31	67	16	43	49.9%	1.45 [0.66, 3.18]
Zahra 2015	9	31	13	41	37.9%	0.88 [0.32, 2.44]
**Subtotal (95% CI)**		**116**		**106**	**100.0%**	**1.10 [0.61, 1.99]**
Total events	41		32			
Heterogeneity: Chi² = 1.48, df = 2 (P = 0.48); I² = 0%						
Test for overall effect: Z = 0.33 (P = 0.74)						

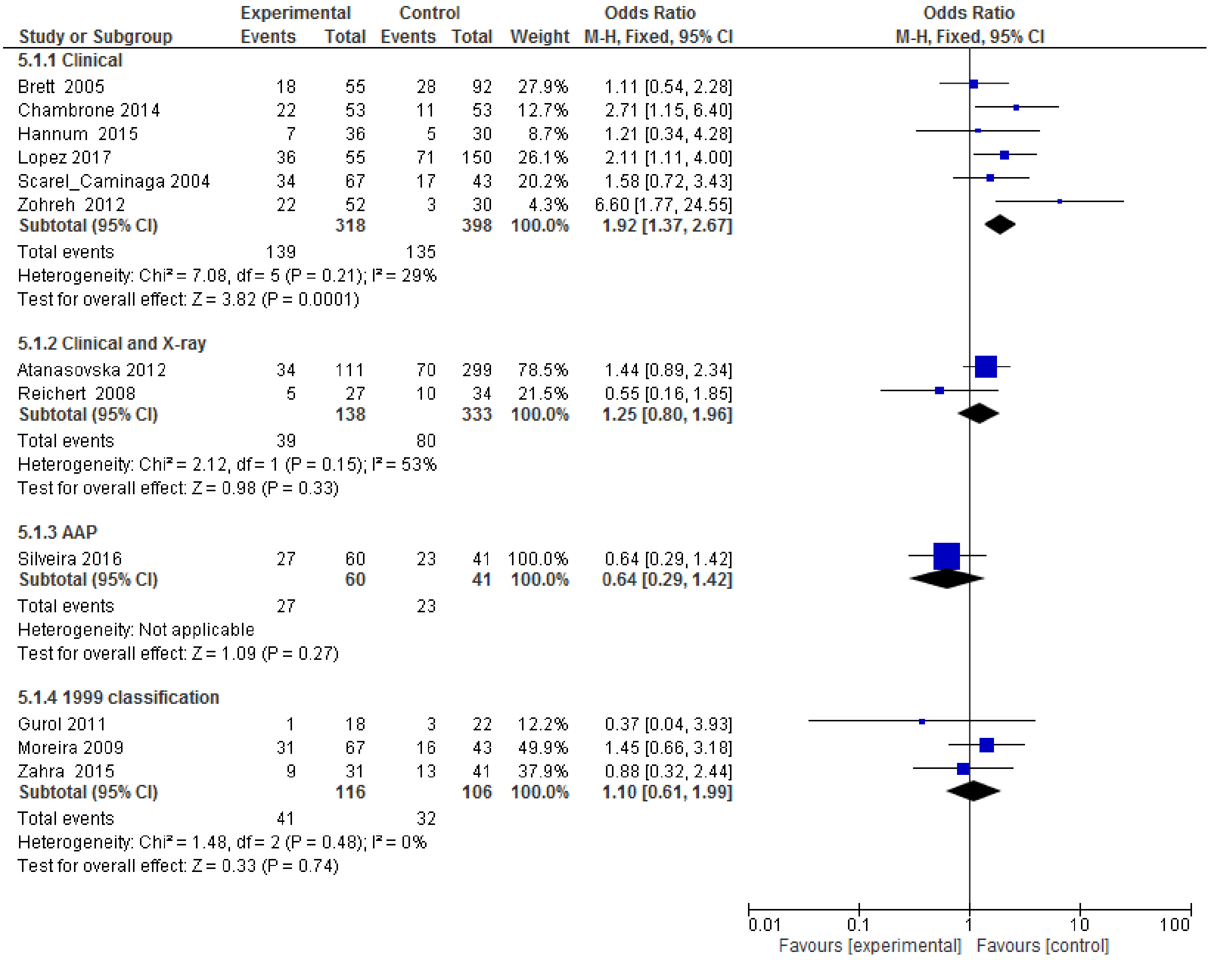

Clinical: Clinical diagnosis; Clinical & X-ray: Clinical diagnosis, followed by X-ray verification;

1999 classification: 1999 classification of periodontal diseases and conditions; AAP: American Academy of Periodontology criteria.

The boxes show the effect estimates from the individual studies, while the diamond shows the pooled result.

The I^2 value 0 % indicates an absence of heterogeneity. A fixed effect model was used in the absence of heterogeneity.
