# Supplementary figures and images for: The role of three interleukin 10 gene polymorphisms (− 1082 A > G, − 819 C > T, − 592 A > C) in the risk of chronic and aggressive periodontitis: a meta-analysis and trial sequential analysis
Source: BMC Oral Health. 2018 Oct 22;18:171. doi: 10.1186/s12903-018-0637-9 (PMC6198364; doi:10.1186/s12903-018-0637-9)

Additional File 7. Funnel plot of comparison: TNF 308 -1082 A>G dominant model

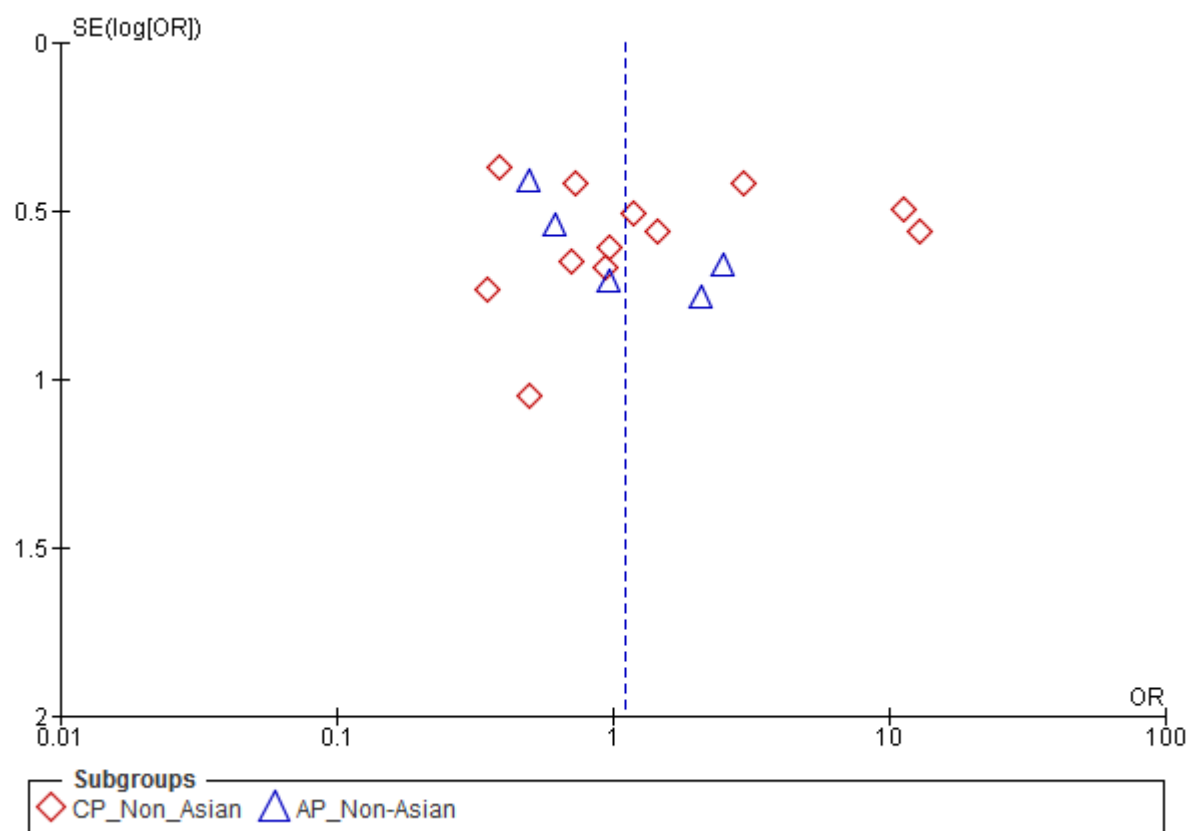

Supplement: Supplementary file 7 — Funnel plot of studies on IL10–1082 A > G in the recessive model. (PDF 84 kb) [file 12903_2018_637_MOESM7_ESM.pdf]
