# Supplementary material for: The role of three interleukin 10 gene polymorphisms (− 1082 A > G, − 819 C > T, − 592 A > C) in the risk of chronic and aggressive periodontitis: a meta-analysis and trial sequential analysis
Source: BMC Oral Health. 2018 Oct 22;18:171. doi: 10.1186/s12903-018-0637-9 (PMC6198364; doi:10.1186/s12903-018-0637-9)
Supplement: Supplementary file 8 — Trial sequential analysis of studies on IL10–1082 A > G in chronic periodontitis. (PDF 211 kb) [file 12903_2018_637_MOESM8_ESM.pdf]

Additional File 8. Trial sequential analysis of studies on 1082 A>G in chronic periodontitis

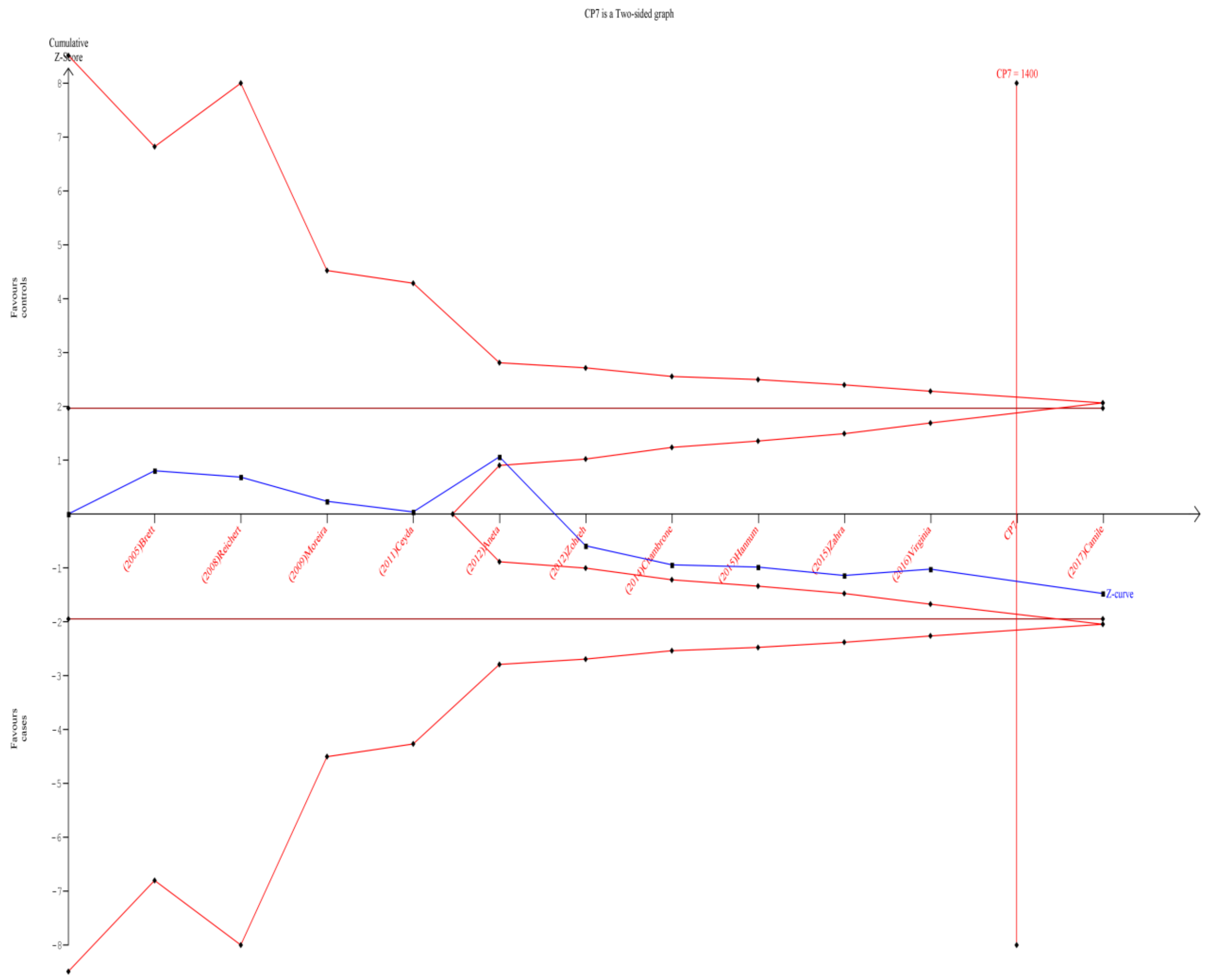

The cumulative z-curve (blue line) crossed the traditional boundary and the trial sequential monitoring boundary and reached the required information size, suggesting there is no need for more evidence to establish additional study of -1082 A>G in CP. Horizontal lines: conventional boundaries (upper for benefit, Z-score = 1.96, lower for harm, Z-score = −1.96, two-sided P = 0.05); Sloping red full lines with black square fill icons: trial sequential monitoring boundaries calculated accordingly; Vertical red full line: required information size calculated accordingly.
